# Hope in Miniature: The First Case of Implantation of a “Tiny Pacemaker” in Italy as a Successful Treatment for Congenital Atrioventricular Block in a Low Birth Weight Child

**DOI:** 10.1155/cric/3682992

**Published:** 2026-01-20

**Authors:** Ferrari Paola, Limonta Raul, Malanchini Giovanni, Patanè Luisa, De Filippo Paolo

**Affiliations:** ^1^ Cardiac Electrophysiology Unit, ASST Papa Giovanni XXIII, Bergamo, Italy, asst-pg23.it; ^2^ School of Medicine and Surgery, University of Milano-Bicocca, Milan, Italy, unimib.it; ^3^ Obstetrics and Gynecology Unit, ASST Papa Giovanni XXIII, Bergamo, Italy, asst-pg23.it

## Abstract

Congenital complete atrioventricular block (CAVB) is a rare cardiac condition occurring in approximately one in 15,000 to one in 22,000 live births. Maternal autoimmune diseases, with anti‐ssA (Ro) and anti‐ssB (La) antibodies implicated in 56%–90% of cases, are primary causes. We present a case of a 31‐year‐old primigravid woman referred at 29 weeks of gestation for fetal high‐grade AVB, initially diagnosed as 2:1 AVB with a ventricular rate of 45 bpm. Maternal corticosteroid therapy was initiated for suspected immune‐mediated etiology, pending autoantibody test results. Upon transfer, a 3:1 AVB was detected, with fetal heart failure signs. Genetic and autoimmune evaluations ruled out primary electrical heart diseases and infections. Prompt intervention was necessitated due to fetal cardiac decompensation. Sympathomimetic drugs via placental circulation were ineffective. Cesarean section was scheduled at 30 weeks and 1 day. The neonate, weighing 1280 g, had an APGAR score of 7 and a heart rate of 40 bpm. Initial resuscitation and isoproterenol infusion resulted in a moderate heart rate increase. Temporary pacing wires were surgically placed. As permanent pacemaker implantation became necessary, traditional venous access was impractical. A “tiny pacemaker” made with modification of a Medtronic Micra MC1VR01 generator connected to an epicardial lead ensured hemodynamic stability. Approval of this off‐label device from the Italian Ministry of Health was swiftly obtained. Diagnosis of CAVB typically involves fetal echocardiography and fetal magnetocardiography for precise arrhythmia diagnosis. Treatment varies, with fluorinated steroids reducing block severity in autoimmune cases. Miniaturized pacemakers offer a promising solution for neonates, addressing challenges of conventional devices. Further research is needed to evaluate their long‐term efficacy and safety, potentially benefiting patients with venous and cardiac abnormalities.

## 1. Case Presentation

We present the first case in Europe of a child with idiopathic congenital high‐grade atrioventricular block (AVB) resulting in biventricular heart failure and fetal hydrops, effectively managed by implanting a prototype of permanent “tiny pacemaker,” following maternal delivery at 30 weeks of gestation.

No written consent has been obtained from the patient, as there is no patient‐identifiable data included in this case report.

### 1.1. Background

A 31‐year‐old primigravid woman (G1P0) was referred to our specialized center for pathological pregnancies during her 29th week of gestation, following the identification of high‐grade AVB. The initial rhythm of presentation of the fetus was 2:1 AVB with an average ventricular rate of 45 bpm, and this was diagnosed during a second‐trimester obstetrical ultrasound conducted at another facility 5 weeks earlier.

Until that point, the pregnancy had proceeded uneventfully with appropriate biometry for gestational age and prenatal screening tests including noninvasive prenatal testing (NIPT) and nuchal translucency (NT), which yielded negative results for chromosomal abnormalities. Given the suspicion of an immune‐mediated etiology and concern regarding potential progression to congenital complete atrioventricular block (CAVB), the pregnant lady was admitted to the hospital and maternal corticosteroid therapy with dexamethasone (8 mg orally once daily) was cautiously initiated while awaiting the results of maternal autoantibody testing, specifically targeting antinuclear antibodies (ANAs), anti‐ssA (Ro), and anti‐ssB (La).

Upon transfer to our center, a subsequent obstetric ultrasound revealed persistent high‐grade AVB, now at a 3:1 ratio with an average heart rate of 35 bpm to a nadir of 20 bpm, along with previously absent signs of fetal cardiac decompensation. Notably, at fetal cardiac ultrasound, both the left and right ventricles appeared dilated and diffusely moderately hypokinetic, along with modest circumferential pericardial effusion and ascites.

Considering the negative results of the autoimmune laboratory tests conducted on the mother and the ineffectiveness of corticosteroid therapy in halting the disease progression and restoring normal atrioventricular (AV) conduction, the hypothesis of an immune‐mediated etiology was ruled out. In search of an alternative cause, both parents underwent comprehensive clinical and genetic evaluation, particularly to screen for primary electrical heart diseases such as CAVB and long QT syndrome (LQTS), yielding negative results. Moreover, maternal hypothyroidism and pregnancy‐related infections were excluded as potential causing factors.

An echocardiographic assessment conducted by expert pediatric cardiologists confirmed the presentation of fetal heart failure secondary to severe bradyarrhythmia in an otherwise structurally normal heart. In this scenario, prompt intervention was warranted to prolong the gestational period and try to postpone delivery. In this regard, we attempted to infuse sympathomimetic drugs into the fetus utilizing the placental circulation to elicit a positive dromotropic effect. Initially, ritodrine was infused with no or very limited effect on heart rate; then, we decided to infuse isoproterenol, which exerted no effect on AV conduction but increased only maternal heart rate. The fetal heart rhythm was further confirmed by fetal magnetocardiography (fMCG) at 30 weeks gestation, revealing an average fetal ventricular rate of 35–40 bpm.

Potential risks associated with prematurity in the context of a hydropic newborn were discussed to facilitate parental decision‐making regarding delivery, and the parents accepted.

Given the ineffectiveness of pharmacological treatment, in order to prevent further deterioration of the fetal clinical condition and subsequent intrauterine death, it became necessary to schedule a cesarean section, which was programmed with the presence of a cardiothoracic surgeon expert in congenital heart disease stand‐by.

The delivery was carried out at 30 weeks and 1 day of gestation, with the neonate weighing 1280 g and measuring 39 cm. The infant presented with an APGAR score of 7 points at 1 min, accompanied by a mean heart rate of 40 bpm and suboptimal pulse oximetry. Immediate pediatric resuscitation measures were implemented but failed to improve both heart rate and oxygen saturation. Following the placement of a peripheral venous access, an infusion of isoproterenol (0.02 mcg/kg/min) was initiated, resulting in limited efficacy. Subsequently, umbilical access was secured to administer higher doses of the sympathomimetic agent (isoproterenol up to 0.6 mcg/kg/min), leading to a moderate but still unsatisfactory increase in heart rate to approximately 70 bpm. In order to maintain adequate cardiac output and, therefore, peripheral perfusion, adrenaline was introduced and titrated from 0.1 up to 0.3 mcg/kg/min. As predicted, surgical placement of two pairs of temporary pacing wires, one for the atria and the other for the ventricles, programmed in VVI mode at a rate of 100 bpm, was deemed necessary, allowing for the gradual tapering of isoproterenol infusion rate until its discontinuation and a consensual increase of the pacing rate up to 120 bpm.

Due to multiple unsuccessful attempts at discontinuing epicardial stimulation and the progressive increase in lead thresholds, it was deemed necessary to implant a permanent pacemaker as soon as possible. However, infants pose several challenges, particularly regarding the impracticality of venous access for leads. Consequently, epicardial stimulation using a tunneled bipolar lead placed in the abdomen emerged as the preferred treatment. However, in infants weighing less than 2.5 kg, the generator′s size incongruity may cause adverse local effects such as wound dehiscence and skin erosion. Therefore, a decision was made to explore a novel technology, as outlined in a recent series of five clinical cases. We implanted a prototype “tiny pacemaker” where a Medtronic Micra MC1VR01 generator was incorporated into a polymer header to enable connection with the CapSure Epi model 4968 bipolar IS‐1 epicardial lead (Medtronic Inc.), specifically designed for this unique neonatal population. This device was obtained by the manufacturer but does not have regulatory approval. So, a compassionate use can be authorized by local authorities. The surgical technique used was a standard abdominal pocket creation (with smaller size) with subxiphoid section and connection to the epicardial ventricular lead.

The device was programmed in VVI mode at 100 bpm, effectively maintaining hemodynamic stability.

Due to the absence of prior Italian and European cases, approval from our “Ministero della Salute” was required; despite the intricate Italian bureaucratic system, approval for the procedure was obtained just 3 days later, given the exceptional nature and urgency of this case, after consultation with our local ethical committee in the Hospital. In such a process, the understanding of the extraordinary needs of this very small patient was thoroughly explained to both parents, who signed consent and authorization for the implantation of an off‐label device.

At the time of discharge, growth was satisfactory (2892 g, 43 cm), along with hemodynamic parameters and pacemaker function.

### 1.2. Follow‐Up

**Figure 1:**
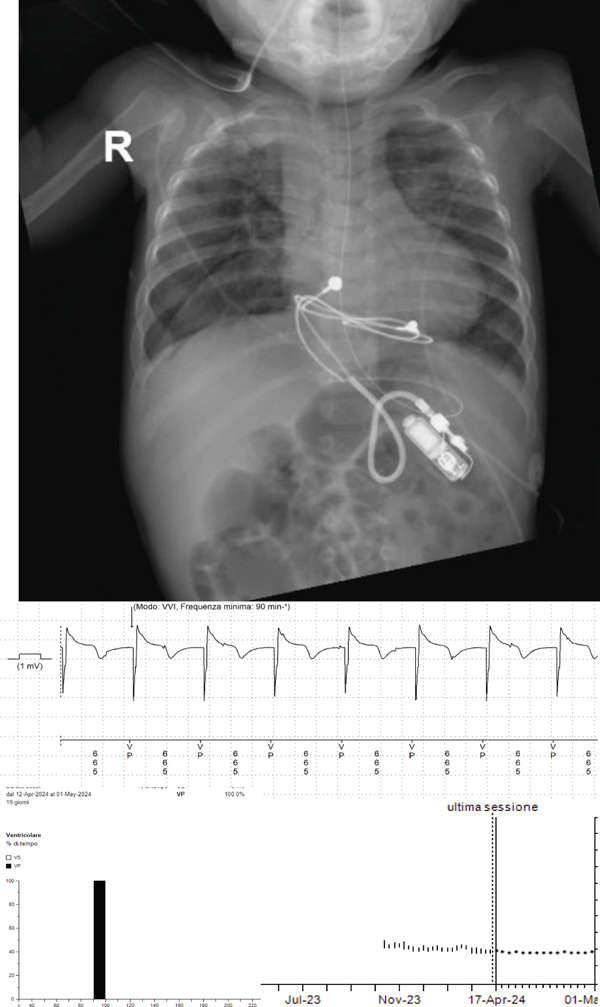
Chest x‐ray showing tiny pacemaker implant and pacemaker charts showing stable parameters and 100% ventricular pacing at follow‐up.

The first cardiology follow‐up, approximately 3 months later, revealed normal cardiac function and good electrical parameters of the implanted pacemaker, with a pacing percentage of 99.7% (see Figure 1).

After implantation of this epicardial lead at the age of 6 months, our patient was admitted to the hospital for viral pneumonia. The device check showed a slightly increased threshold for ventricular pacing at that time. At the age of approximately 9 months, a second lead was implanted due to fracture of the first one and connected to a standard single chamber device, now in a baby with a weight that was over 6 kg.

## 2. Discussion

### 2.1. Epidemiology and Etiology

CAVB is a rare condition occurring in approximately 1/15,000 to 1/22,000 live births [1], primarily attributed to autoimmune etiologies, which account for 56%–90% of cases [2]. This form is characterized by the absence of anatomical malformations in the heart, accompanied by maternal autoimmune disease and/or autoantibody positivity, particularly anti‐ssA (Ro) and anti‐ssB (La) antibodies. This inflammatory process typically reaches its peak between 18 and 25 weeks of gestation [3], although diagnosis may be delayed until after birth in some instances. While maternal antibodies are detectable in 95% of mothers of infants with isolated CAVB, only 20%–30% of infants with CAVB have mothers with a previous autoimmune disease diagnosis, and most are asymptomatic [4]. The second most prevalent cause of CAVB is congenital heart disease, affecting 14%–42% of cases and carrying the poorest prognosis [2]. This condition is attributed to defects in cardiac embryogenesis that result in impaired electrical signal propagation and an increased predisposition to CAVB. Structural anomalies such as transposition of the great vessels (TGV) and defects of the AV septum are also common, with approximately 5% of TGV cases diagnosed at birth exhibiting complete CAVB [5]. Additionally, idiopathic CAVB, characterized by genetic abnormalities unrelated to autoimmune disease and occurring in a structurally normal heart, is recognized [5]. Moreover, notable risk factors for CAVB include maternal infections and hypothyroidism during pregnancy, which must always be ruled out. A prior study documented fetal mortality with intrauterine hydrops and fetal demise in 7% of their cohort, with an additional 10%–15% mortality during infancy alone [6]. Prematurity also confers additional risk, with complications including the need for inotropic support and temporary pacing while awaiting permanent pacemaker placement [7]. Poorer overall perinatal outcomes have been associated with lower ventricular rates less than 50 bpm, a rapidly decreasing fetal heart rate, and other findings indicative of poor ventricular function, AV valve regurgitation, or hydrops [8].

### 2.2. Diagnosis

The diagnosis of CAVB is typically established via fetal echocardiography, which offers a comprehensive evaluation of fetal cardiac anatomy and function, encompassing rhythm and rate assessment by analyzing atrial and ventricular rates, AV conduction, and ventricular contractility following atrial contractions [9]. Moreover, pulsed wave Doppler echocardiography further aids in evaluating flow across cardiac valves to demonstrate the dissociation between atrial and ventricular flows [10]. While not as widely accessible, fMCG offers superior precision in diagnosing fetal arrhythmia, guiding medical therapy adjustments, and informing delivery planning [11].

### 2.3. Treatment

Bearing in mind the principle of “primum non nocere,” treatment approaches for CAVB vary widely, lacking consensus within the medical community. Its selection hinges upon the underlying cause of the AVB, ventricular function, and the severity of heart failure. Fluorinated steroids have primarily been utilized in cases of immune‐mediated CAVB (Class IIb, Level C) [9]. Some studies have advocated for oral dexamethasone (or betamethasone) at doses ranging from 4 to 8 mg/ day for a duration of 6 weeks [2, 12, 13]. This treatment regimen should be continued until delivery if reversal of the AVB is achieved. However, it should be discontinued if the AVB persists, or if there is evidence of fetal hydrops, even in cases of complete AVB [14]. These medications lessen the severity of the block and reduce the need for pacemaker implantation. While their efficacy in cases of irreversible complete CAVB has not been demonstrated, they may be utilized temporarily in situations of uncertainty regarding the degree of block, as was the case with this patient. In these circumstances, sympathomimetics are favored for elevating fetal heart rate, especially when it drops below 50–55 bpm, to improve both heart rate and cardiac output [9, 15, 16]. However, no studies have demonstrated improved survival rates with their use [17]. In addition, potential side effects including anxiety, palpitations, and headache may limit maternal tolerance. Planning for delivery and neonatal resuscitation is crucial due to the risk of hemodynamic instability resulting from bradycardia‐induced poor systemic perfusion. The primary objective is to optimize arterial oxygen saturation, increase neonatal heart rate, and ensure adequate systemic perfusion during transport from the delivery to the cardiac operating room. In order to facilitate these urgent interventions, neonatal care should be arranged at a tertiary care center. Upon birth, fetal bradycardia, which is defined by a heart rate less than 70 bpm, can be managed initially with adrenergic receptor agonists such as isoprenaline, epinephrine, and/or dopamine, either singly or in combination with temporary cardiac pacing, especially in cases of fetal hydrops or cardiogenic shock [15–18]. Unfortunately, CAVB is irreversible in approximately 80% of cases, necessitating subsequent permanent pacemaker implantation, which has been demonstrated to improve long‐term survival even in asymptomatic cases [19]. However, pacemaker implantation in neonate patients presents technical challenges, compounded by their small size, rapid growth, and the size discrepancy between the device, and the child′s body complicates the procedure. Moreover, the transvenous route is not a viable option and epicardial pacing with placement of the pacemaker generator in the abdominal wall becomes the preferred method according to current guidelines [15, 16, 20, 21]. In the neonatal population, particularly among preterm infants of less than 2.5 kg, the size of conventional generators presents a notable risk of wound dehiscence, generator‐related skin erosion, and other local complications, making their use inconvenient. A novel miniaturized generator (29.4 × 16.6 × 9.6 mm, 3.5 cc, 5 g) developed by Medtronic Inc. offers a potential solution to this challenge by enabling the connection of standard epicardial pacing leads to a tiny implantable pulse generator, effectively accounting for all the aforementioned issues. However, this solution also presents some limitations, including the significantly shorter longevity, most likely due to a combination of higher pacing rates in neonates and higher epicardial thresholds, resulting in higher programmed outputs. Furthermore, there is an ongoing debate regarding the ideal pacing mode [22], and VVI mode remains a common preference in smaller children at the outset [23–26], with the rate‐responsive function being of limited significance until the neonate becomes physically active. This strategy may not only reduce the risks associated with permanent pacemaker implantation in neonates but can also be beneficial for patients unable to undergo conventional implantation strategies due to factors such as patient size, venous abnormalities, and congenital heart disease [27, 28]. Miniaturized or leadless pacemakers represent a significant technological advance over conventional epicardial and transvenous systems in neonates and infants. The absence of transvenous leads eliminates the risk of venous thrombosis, stenosis, or lead fracture—events that are particularly concerning in patients with low body weight or limited vascular access. The recent multicenter experience by Berul et al. [29] in 29 infants (median age 15 days, 2.3 kg) confirmed the procedural feasibility and electrical stability of a novel pediatric pacemaker, with no device‐related deaths and only isolated complications mainly due to infection or growth‐related replacement. In contrast, conventional epicardial pacing systems in this population have been associated with infection, lead dislodgement, or generator erosion in up to 25% of cases. Moreover, the miniaturized generator allows implantation through a smaller surgical pocket—often subxiphoid or subrectus—reducing wound dehiscence, postoperative pain, and recovery time. From a long‐term perspective, these devices can provide an effective bridge strategy until somatic growth permits implantation of a standard pacemaker, minimizing early surgical trauma and improving quality of life in “tiny patients.” Nevertheless, long‐term durability, extraction strategies, and battery longevity remain to be fully defined, warranting ongoing follow‐up and multicenter surveillance.

Miniature pacemaker technology extends beyond neonates with irreversible CAVB, offering a versatile option across multiple age groups and clinical contexts: These include symptomatic bradyarrhythmia secondary to congenital heart disease, persistent postoperative AVB, sinus node dysfunction with clinically significant pauses, and hemodynamic instability related to conduction disturbances, with or without structural abnormalities [29]. Approximately one in four neonates eligible for miniature pacing presents with structural heart disease, and implantation may occasionally be prophylactic to prevent sudden death or progressive ventricular dysfunction [29]. Beyond neonates, potential candidates include pediatric patients with limited venous access or complex congenital anatomy, adults at high risk of infection, those with very low body weight, and scenarios requiring temporary or bridging pacing due to device infection or critical illness.

### 2.4. Global Access and Policy Implications

The widespread adoption of miniature or leadless pacemaker systems in neonates and infants remains constrained by regulatory, economic, and infrastructural factors, particularly across Europe. Regulatory heterogeneity, lack of specific reimbursement frameworks, and the high upfront cost of these devices continue to pose major barriers. The cost of neonatal miniature pacemakers is significantly higher than that of conventional generators, not only because of the device itself but also due to the need for customized implantation techniques and specialized surgical expertise. Implementation is further limited by the scarcity of centers experienced in epicardial miniature implantation, the requirement for dedicated surgical materials, and the logistical burden of intensive follow‐up, including potential reinterventions for somatic growth and generator replacement [29]. To promote equitable and sustainable access, several coordinated strategies such as pilot programs may accelerate the global dissemination of miniature pacemaker technologies, ensuring that innovations in device miniaturization translate into tangible clinical benefits for infants and children across diverse healthcare systems.

## 3. Conclusions

Unlike in the United States [28, 29], “tiny pacemakers” have not yet been implemented in clinical practice in Europe. However, as shown by our case report, they may have a role in neonates with irreversible CAVB who require permanent epicardial pacing, addressing limitations related to the size of conventional generators, which is one of the many unmet clinical needs in these “tiny patients.” Additionally, these devices might also be applicable in specific other populations, such as those with venous and cardiac abnormalities that hinder transvenous and endocardial lead placement. Priority areas for research include prospective cohorts′ registries with extended follow‐up to assess device longevity, need for revisions, and long‐term clinical outcomes. Comparative studies between leadless and conventional epicardial approaches are also warranted when feasible.

## Conflicts of Interest

The authors declare no conflicts of interest.

## Funding

No funding was received for this manuscript.

## Data Availability

The sensitive data of this patient is stored on our local servers and can be disclosed to the public with the consent of the patient and/or their family members.
